# On $\frac{1}{w} + \frac{1}{x} + \frac{1}{y} + \frac{1}{z} = \frac{1}{ 2} $ and some of its generalizations

**DOI:** 10.1186/s13660-018-1782-z

**Published:** 2018-07-28

**Authors:** Tingting Bai

**Affiliations:** College of Mathematics and Information Science, Baoji University of Arts and Sciences, Baoji, China

**Keywords:** 11D68, Diophantine equation, Integer solution

## Abstract

In this paper, we give a straightforward approach to obtaining the solution of the Diophantine equation $\frac{1}{w} + \frac{1}{x} + \frac{1}{y} + \frac{1}{z} = \frac{1}{2}$. We also establish that the Diophantine equation $\frac{1}{w} + \frac{1}{x} + \frac{1}{y} + \frac{1}{z} = \frac{m}{n}$ for any two positive integers *m* and *n* has only a finite number of solutions in the positive integers $w, x, y$, and *z*.

## Introduction and preliminaries

The unit fractional decomposition of certain rational fractions was considered one of fascinating problems by the ancient Egyptians. One of such problems is a well-known conjecture due to Erdos and Strauss in 1948. They conjectured that for each $n>1$, the Diophantine equation

$$\frac{4}{n} = \frac{1}{x} + \frac{1}{y} + \frac{1}{z} $$

has a solution in positive integers $x, y$, and *z*. Although it has been investigated by many mathematicians, the conjecture is still open. A good number of partial results have been obtained by several mathematicians (see [1, 3, 5, 6, 8, 9]). Mordell [7] has proven that the conjecture is true for all *n* except possibly cases in which *n* is congruent to $1, 121, 169, 289, 361, 529\ ( \mathrm{mod}\ 840)$. For the extensive literature +Sierpinski, Schinzel, and others, we refer the reader to [4]. Recently, Elsholtz and Tao [2] investigated the average behavior of a number of positive integer solutions in $x, y$, and *z* of the above Diophantine equation in the case when *n* is prime.

In this paper, we consider an analogue of the above conjecture of Erdos and Strauss. More precisely, we study the Diophantine equation

1.1 $$ \frac{1}{w} + \frac{1}{x} + \frac{1}{y} + \frac{1}{z} = \frac{1}{ 2} $$

and give a detailed solution to Eq. (1.1). We also draw our attention to some of the generalizations of Eq. (1.1). We use elementary arguments and inequalities to prove the results.

## Main results and discussion

In this section, we first find the solutions in positive integers $x, y, z$, and *w* of Eq. (1.1).

Without loss of generality, we may assume that $w\leq x\leq y\leq z$. Then Eq. (1.1) gives:

- $\frac{1}{w} < \frac{1}{2} $ and thus $w\geq3$;
- $\frac{1}{w} + \frac{1}{x} + \frac{1}{y} + \frac{1}{z} \geq \frac{4}{z}$ and thus $z \geq 8$; and
- $\frac{1}{w} + \frac{1}{x} + \frac{1}{y} + \frac{1}{z} \leq \frac{4}{w}$ and thus $w\leq8$.

Using (a) and (c), we see that $w\in\{3, 4, 5, 6, 7, 8\}$. Thus Eq. (1.1) can be rewritten as follows:

When $w=3$,

2.1 $$\begin{aligned} \frac{1}{x} + \frac{1}{y} + \frac{1}{z} = \frac{1}{6}, \end{aligned}$$

When $w=4$,

2.2 $$ \frac{1}{x} + \frac{1}{y} + \frac{1}{z} = \frac{1}{4}, $$

When $w=5$,

2.3 $$ \frac{1}{x} + \frac{1}{y} + \frac{1}{z} = \frac{3}{10}, $$

When $w=6$,

2.4 $$ \frac{1}{x} + \frac{1}{y} + \frac{1}{z} = \frac{1}{3}, $$

When $w=7$,

2.5 $$ \frac{1}{x} + \frac{1}{y} + \frac{1}{z} = \frac{5}{14}, $$

When $w=8$,

2.6 $$ \frac{1}{x} + \frac{1}{y} + \frac{1}{z} = \frac{3}{8}. $$

We now find the solution of Eq. (2.1).

Clearly $\frac{1}{x} < \frac{1}{6}$ and thus $x>6$.

Under the assumption $x\leq y\leq z$, Eq. (2.1) gives $\frac{1}{x} + \frac{1}{y} + \frac{1}{z} \leq \frac{3}{x}$ and thus $x\leq18$.

Hence $x\in \{ 7, 8, 9, 10, 11, 12, 13, 14, 15, 16, 17, 18 \}$, and thus we have the following cases:

For $x=7$,

2.7 $$ \frac{1}{y} + \frac{1}{z} = \frac{1}{42}, $$

For $x=8$,

2.8 $$ \frac{1}{y} + \frac{1}{z} = \frac{1}{24}, $$

For $x=9$,

2.9 $$ \frac{1}{y} + \frac{1}{z} = \frac{1}{18}, $$

For $x=10$,

2.10 $$ \frac{1}{y} + \frac{1}{z} = \frac{1}{15}, $$

For $x=11$,

2.11 $$ \frac{1}{y} + \frac{1}{z} = \frac{5}{66}, $$

For $x=12$,

2.12 $$ \frac{1}{y} + \frac{1}{z} = \frac{1}{12}, $$

For $x=13$,

2.13 $$ \frac{1}{y} + \frac{1}{z} = \frac{7}{78}, $$

For $x=14$,

2.14 $$ \frac{1}{y} + \frac{1}{z} = \frac{2}{21}, $$

For $x=15$,

2.15 $$ \frac{1}{y} + \frac{1}{z} = \frac{1}{10}, $$

For $x=16$,

2.16 $$ \frac{1}{y} + \frac{1}{z} = \frac{5}{48}, $$

For $x=17$,

2.17 $$ \frac{1}{y} + \frac{1}{z} = \frac{11}{102}, $$

For $x=18$,

2.18 $$ \frac{1}{y} + \frac{1}{z} = \frac{1}{9}. $$

Equations (2.7), (2.8), (2.9), (2.10), (2.12), (2.14), (2.15), (2.18) may be written as follows:

2.7′ $$\begin{aligned} &(y-42) (z-42) = 1764, \end{aligned}$$

2.8′ $$\begin{aligned} &(y-24) (z-24) =576, \end{aligned}$$

2.9′ $$\begin{aligned} &(y-18) (z-18) = 324, \end{aligned}$$

2.10′ $$\begin{aligned} &(y-15) (z-15) = 225, \end{aligned}$$

2.12′ $$\begin{aligned} &(y-12) (z-12) = 144, \end{aligned}$$

2.15′ $$\begin{aligned} &(y-10) (z-10) = 100, \end{aligned}$$

2.18′ $$\begin{aligned} &(y-9) (z-9) = 81. \end{aligned}$$

Under the assumption $x\leq y\leq z$, Eq. (2.1) gives $\frac{1}{x} + \frac{1}{y} + \frac{1}{z} \geq \frac{3}{z}$ and thus

2.13 $$ z\geq18. $$

Under inequality (2.13) and $(y-42)\leq(z-42)$, Eq. (2.7′) leads to:

$$\begin{aligned} &(y-42)=1,\qquad (z-42)=1764, \\ &(y-42)=2,\qquad (z-42)=882, \\ &(y-42)=3,\qquad (z-42)=588, \\ &(y-42)=4,\qquad (z-42)=441, \\ &(y-42)=6,\qquad (z-42)=294, \\ &(y-42)=7,\qquad (z-42)=252, \\ &(y-42)=9,\qquad (z-42)=196, \\ &(y-42)=12,\qquad (z-42)=147, \\ &(y-42)=14,\qquad (z-42)=126, \\ &(y-42)=18,\qquad (z-42)=98. \end{aligned}$$

Thus $( y, z ) \in \{ ( 43, 1806 ), ( 44, 924 ), ( 45, 630 ), ( 46,483 ), ( 48, 336 ), ( 49, 294 ), ( 51, 238 ), ( 54, 189 ), (56, 168), ( 60, 140 ) \}$.

Hence Eq. (2.7′) leads to the following solutions of Eq. (1.1):

$$\begin{aligned} ( w, x, y, z ) \in{}& \bigl\{ ( 3, 7, 43, 1806 ), ( 3, 7, 44, 924 ), ( 3, 7, 45, 630 ), ( 3, 7, 46,483 ), ( 3, 7, 48, 336 ), \\ & ( 3, 7, 49, 294 ), ( 3, 7, 51, 238 ), ( 3, 7, 54, 189 ), (3, 7, 56, 168), ( 3, 7, 60, 140 ) \bigr\} . \end{aligned}$$

Under inequality (2.13) and $(y-24)\leq(z-24)$, Eq. (2.8′) gives the following solutions:

$$\begin{aligned} ( y, z ) ={}&\bigl\{ ( 25, 600 ), ( 26, 312 ), ( 27, 216 ), ( 28, 168 ), ( 30, 120 ), ( 32, 96), (33, 88 ), ( 36, 72 ), \\ & ( 40, 60 ), (42, 56)\bigr\} . \end{aligned}$$

Hence Eq. (2.8′) leads to the following solutions of Eq. (1.1):

$$\begin{aligned} ( w, x,y, z ) \in{}&\bigl\{ ( 3, 8, 25, 600 ), ( 3, 8, 26, 312 ), ( 3, 8, 27, 216 ), ( 3, 8, 28, 168 ), ( 3, 8, 30, 120 ), \\ &( 3, 8, 32, 96), (3, 8, 33, 88 ), ( 3, 8, 36, 72 ), ( 3, 8, 40, 60 ), (3, 8, 42, 56)\bigr\} . \end{aligned}$$

Under inequality (2.13) and $(y-18)\leq(z-18)$, Eq. (2.9′) gives the following solutions:

$$( y,z ) \in\bigl\{ ( 19,342 ), ( 20, 180 ), ( 21,126 ), ( 22, 99 ), ( 24, 72 ), ( 27, 54 ), ( 30, 45 ), (36, 36)\bigr\} . $$

Hence Eq. (2.9′) leads to the following solutions of Eq. (1.1):

$$\begin{aligned} ( w, x,y,z ) \in{}&\bigl\{ ( 3, 9,19,342 ), ( 3, 9,20, 180 ), ( 3, 9,21,126 ), ( 3, 9,22, 99 ), ( 3, 9,24, 72 ), \\ &( 3, 9,27, 54 ), ( 3, 9,30, 45 ), (3, 9,36, 36)\bigr\} . \end{aligned}$$

Under inequality (2.13) and $(y-15)\leq(z-15)$, Eq. (2.10′) gives the following solutions:

$$( y,z ) \in \bigl\{ ( 16, 240 ), ( 18, 90 ), ( 20, 60 ), ( 24, 40 ), ( 30, 30 ) \bigr\} . $$

Hence Eq. (2.10′) leads to the following solutions of Eq. (1.1):

$$( w, x, y,z ) \in \bigl\{ ( 3, 10, 16, 240 ), ( 3, 10, 18, 90 ), ( 3, 10, 20, 60 ), ( 3, 10, 24, 40 ), ( 3, 10, 30, 30 ) \bigr\} . $$

Under inequality (2.13) and $(y-12)\leq(z-12)$, Eq. (2.12′) gives the following solutions:

$$( y,z ) \in \bigl\{ ( 13,156 ), ( 14, 84 ), ( 15,60 ), ( 16,48 ), ( 18,36 ), ( 20, 30 ), ( 21, 28 ), ( 24, 24 ) \bigr\} . $$

Hence Eq. (2.12′) leads to the following solutions of Eq. (1.1):

$$\begin{aligned} ( w, x,y,z ) \in{}&\bigl\{ ( 3, 12, 13,156 ), ( 3, 12, 14, 84 ), ( 3, 12, 15,60 ), ( 3, 12, 16,48 ), ( 3, 12, 18,36 ), \\ &( 3, 12, 20, 30 ), ( 3, 12, 21, 28 ), ( 3, 12, 24, 24 ) \bigr\} . \end{aligned}$$

Under inequality (2.13) and $(y-10)\leq(z-10)$, Eq. (2.15′) gives the following solutions:

$$( y,z ) \in\bigl\{ ( 11,110 ), ( 12, 60 ), ( 14, 35 ), ( 15, 30 ), ( 20,20 ) \bigr\} . $$

Hence Eq. (2.15′) leads to the following solutions of Eq. (1.1):

$$( w,x,y,z ) \in\bigl\{ ( 3,15,11,110 ), ( 3,15,12, 60 ), ( 3,15,14, 35 ), ( 3,15,15, 30 ), ( 3,15, 20,20 ) \bigr\} . $$

Under inequality (2.13) and $(y-9)\leq(z-9)$, Eq. (2.18′) gives the following solutions:

$$( y,z ) \in \bigl\{ ( 10,90 ), ( 12,36 ), ( 18,18 ) \bigr\} . $$

Hence Eq. (2.18′) leads to the following solutions of Eq. (1.1):

$$( w,x,y,z ) \in \bigl\{ ( 3,18, 10,90 ), ( 3,18, 12,36 ), ( 3,18, 18,18 ) \bigr\} . $$

Since $y\leq z$, $\frac{1}{y} + \frac{1}{z} \leq \frac{2}{y}$ and thus Eq. (2.11) gives

$$\frac{5}{66} \leq \frac{2}{y} \quad\Rightarrow\quad y \leq 26. $$

Again we have $y \geq x=11$ and hence $y\in\{11,12,13,\ldots,26\}$. Therefore the solutions of Eq. (2.11) are as follows:

$$( y,z ) \in \bigl\{ ( 14,231 ), ( 15,110 ), ( 22,22 ) \bigr\} . $$

Hence Eq. (2.11) leads to the following solutions of Eq. (1.1):

$$( w,x,y,z ) \in \bigl\{ ( 3,11,14,231 ), ( 3,11,15,110 ), ( 3,11,22,22 ) \bigr\} . $$

Since $y\leq z$, $\frac{1}{y} + \frac{1}{z} \leq \frac{2}{y}$ and thus Eq. (2.13) gives

$$\frac{7}{78} \leq \frac{2}{y}\quad \Rightarrow \quad y \leq 22. $$

Again we have $y \geq x=13$ and hence $y\in\{13,14,\ldots,22\}$. Therefore the solutions of Eq. (2.13) are as follows:

$$( y,z ) = ( 13,78 ). $$

Hence Eq. (2.13) leads to the following solutions of Eq. (1.1):

$$( w,x,y,z ) = ( 3,13,13,78 ). $$

Since $y\leq z$, $\frac{1}{y} + \frac{1}{z} \leq \frac{2}{y}$ and thus Eq. (2.14) gives

$$\frac{2}{21} \leq \frac{2}{y} \quad\Rightarrow\quad y \leq 21. $$

Again we have $y \geq x=14$ and hence $y\in\{14,\ldots,21\}$. Therefore the solutions of Eq. (2.14) are as follows:

$$( y,z ) \in \bigl\{ ( 14,42 ), ( 15, 35 ), ( 21, 21 ) \bigr\} . $$

Hence Eq. (2.14) leads to the following solutions of Eq. (1.1):

$$( w,x,y,z ) \in \bigl\{ ( 3, 14, 14,42 ), ( 3, 14, 15, 35 ), ( 3, 14, 21, 21 ) \bigr\} . $$

Since $y\leq z$, $\frac{1}{y} + \frac{1}{z} \leq \frac{2}{y}$ and thus Eq. (2.16) gives

$$\frac{5}{48} \leq \frac{2}{y} \quad\Rightarrow\quad y \leq 19. $$

Again we have $y \geq x=16$ and hence $y\in\{16, 17,18,19\}$. Therefore the solutions of Eq. (2.16) are as follows:

$$( y,z ) = ( 16,24 ). $$

Hence Eq. (2.16) leads to the following solutions of Eq. (1.1):

$$( w,x,y,z ) = ( 3,16,16,24 ). $$

Since $y\leq z$, $\frac{1}{y} + \frac{1}{z} \leq \frac{2}{y}$ and thus Eq. (2.17) gives

$$\frac{11}{102} \leq \frac{2}{y}\quad \Rightarrow\quad y \leq 18. $$

Again we have $y \geq x=17$ and hence $y\in\{17,18\}$.

This shows that Eq. (2.17) has no integer solution and hence Eq. (1.1) too has no integer solutions.

We now solve Eq. (2.2), that is, Eq. (1.1) when $w =4$.

It is clear that $\frac{1}{x} < \frac{1}{4} \Rightarrow x>4$.

Under the assumption $x\leq y\leq z$, Eq. (2.2) gives $\frac{1}{x} + \frac{1}{y} + \frac{1}{z} \leq \frac{3}{x}$ and thus $x\leq12$.

Hence $x\in\{5,6,7,8,9 10,11, 12\}$ and thus we have the following cases:

For $x=5$,

2.14 $$ \frac{1}{y} + \frac{1}{z} = \frac{1}{20}, $$

For $x=6$,

2.15 $$ \frac{1}{y} + \frac{1}{z} = \frac{1}{12}, $$

For $x=7$,

2.16 $$ \frac{1}{y} + \frac{1}{z} = \frac{3}{28}, $$

For $x=8$,

2.17 $$ \frac{1}{y} + \frac{1}{z} = \frac{1}{8}, $$

For $x=9$,

2.18 $$ \frac{1}{y} + \frac{1}{z} = \frac{5}{36}, $$

For $x=10$,

2.19 $$ \frac{1}{y} + \frac{1}{z} = \frac{3}{20}, $$

For $x=11$,

2.20 $$ \frac{1}{y} + \frac{1}{z} = \frac{7}{44}, $$

For $x=12$,

2.21 $$ \frac{1}{y} + \frac{1}{z} = \frac{1}{6}. $$

Solving Eqs. (2.14)–(2.21) by the above procedure, we get:

$$\begin{aligned} ( x, y,z ) \in{}&\bigl\{ (5,21,420),(2,22,220),(5,24,120),(5,25,100),(5,28,70),(5,30,60), \\ &( 6,13,156 ), ( 6,14,84 ), ( 6,15,60 ), ( 6,16,48 ), ( 6,18,36 ), ( 6,20,30 ), ( 6,21,28 ),\\& ( 6,24,24 ), ( 8,9,72 ), ( 8,10,40 ), ( 8,12,24 ), ( 8,26,20 ), ( 12,7,42 ),\\ & ( 12,8,24 ), ( 12,9,18 ), ( 12,10,15 ),(12,12,12),(7,10,140),(7,12,42),\\ &(7,14,28),(9,9,36),(9,12,18),(10,10,20),(10,12,15) \bigr\} . \end{aligned}$$

We now solve Eq. (2.3), that is, Eq. (1.1) when $w=5$.

We see that $\frac{1}{x} < \frac{3}{10} \Rightarrow x\geq3$.

Under the assumption $x\leq y\leq z$, Eq. (2.3) gives $\frac{1}{x} + \frac{1}{y} + \frac{1}{z} \leq \frac{3}{x}$ and thus $x\leq10$.

Hence $x\in\{3, 4,5,6,7,8,9,10\}$ and thus Eq. (2.3) leads to the following equations:

For $x=3$,

2.22 $$ \frac{1}{y} + \frac{1}{z} =- \frac{1}{10}, $$

For $x=4$,

2.23 $$ \frac{1}{y} + \frac{1}{z} = \frac{1}{20}, $$

For $x=5$,

2.24 $$ \frac{1}{y} + \frac{1}{z} = \frac{1}{10}, $$

For $x=6$,

2.25 $$ \frac{1}{y} + \frac{1}{z} = \frac{2}{15}, $$

For $x=7$,

2.26 $$ \frac{1}{y} + \frac{1}{z} = \frac{11}{70}, $$

For $x=8$,

2.27 $$ \frac{1}{y} + \frac{1}{z} = \frac{7}{40}, $$

For $x=9$,

2.28 $$ \frac{1}{y} + \frac{1}{z} = \frac{17}{90}, $$

For $x=10$,

2.29 $$ \frac{1}{y} + \frac{1}{z} = \frac{1}{5}. $$

We avoid Eq. (2.22) because it leads to negative solutions.

Solving Eqs. (2.23)–(2.29) by the above procedure, we get:

$$\begin{aligned} ( x, y, z ) \in{}&\bigl\{ ( 4,21, 420 ), ( 4, 22, 220 ), ( 4, 24, 120 ), ( 4, 25, 100 ), ( 4, 28, 70 ), ( 4, 30, 60 ), \\ &( 4, 40, 40 ), ( 5,11,110 ), ( 5,12,60 ), ( 5,14,35 ), ( 5,15,30 ), ( 5,20,20 ), ( 10,6,30 ),\\ & ( 10,10,10 ), (6,8,120),(6,9,45),(6,10,30),(6,12,20),(6,15,15),(7,7,70),\\ &(8,8,20)\bigr\} . \end{aligned}$$

We now solve Eq. (2.4), that is, Eq. (1.1) when $w=6$.

It is clear that $\frac{1}{x} < \frac{1}{3} \Rightarrow x >3$.

Under the assumption $x\leq y\leq z$, Eq. (2.4) gives $\frac{1}{x} + \frac{1}{y} + \frac{1}{z} \leq \frac{3}{x}$ and thus $x\leq9$.

Hence $x\in\{4,5,6,7,8,9\}$ and thus Eq. (2.4) leads to the following equations:

For $x=4$,

2.30 $$ \frac{1}{y} + \frac{1}{z} = \frac{1}{12}, $$

For $x=5$,

2.31 $$ \frac{1}{y} + \frac{1}{z} = \frac{2}{15}, $$

For $x=6$,

2.32 $$ \frac{1}{y} + \frac{1}{z} = \frac{1}{6}, $$

For $x=7$,

2.33 $$ \frac{1}{y} + \frac{1}{z} = \frac{4}{21}, $$

For $x=8$,

2.34 $$ \frac{1}{y} + \frac{1}{z} = \frac{5}{24}. $$

Solving Eqs. (2.30)–(2.26) by the above procedure, we get:

$$\begin{aligned} ( x,y,z ) \in{}&\bigl\{ (4,13,156),(4,14,84),(4,15,60),(4,16,48),(4,18,36),(4,20,30),(4,21,28), \\ &( 6,7,42 ), ( 6,8,24 ), ( 6,9,18 ), ( 6,10,15 ), ( 6,12,12 ), ( 6,15,10 ), ( 5,8,120 ),\\ & ( 5,9,45 ), ( 5,10,30 ), (5,12,20),(5,15,15), (7,7,21),(8,8,12),(8,12,8),\\ &(8,24,6)\bigr\} . \end{aligned}$$

We now solve Eq. (2.5), that is, Eq. (1.1) when $w=7$.

It is clear that $\frac{1}{x} < \frac{5}{14} \Rightarrow x\geq2$.

Under the assumption $x\leq y\leq z$, Eq. (2.5) gives $\frac{1}{x} + \frac{1}{y} + \frac{1}{z} \leq \frac{3}{x}$ and thus $x\leq8$.

Hence $x\in\{2, 3,4,5,6,7,8\}$ and thus Eq. (2.5) leads to the following equations:

For $x=2$,

2.35 $$ \frac{1}{y} + \frac{1}{z} =- \frac{1}{7}, $$

For $x=3$,

2.36 $$ \frac{1}{y} + \frac{1}{z} = \frac{1}{42}, $$

For $x=4$,

2.37 $$ \frac{1}{y} + \frac{1}{z} = \frac{3}{28}, $$

For $x=5$,

2.38 $$ \frac{1}{y} + \frac{1}{z} = \frac{11}{70}, $$

For $x=6$,

2.39 $$ \frac{1}{y} + \frac{1}{z} = \frac{4}{21}, $$

For $x=8$,

2.40 $$ \frac{1}{y} + \frac{1}{z} = \frac{3}{14}. $$

We avoid Eq. (2.35) because it leads to negative solutions.

Solving Eqs. (2.36)–(2.40) by the above procedure, we get:

$$\begin{aligned} ( x, y, z ) \in{}&\bigl\{ (3,43,1806),(3,44,924),(3,45,630),(3,48,483),(3,49,294),(6,6,42), \\ &(6,7,21),(6,9,18), (6,10,15),(6,12,12),(4,10,140),(4,12,42),\\ &(4,14,28),(5,7,70) \bigr\} . \end{aligned}$$

Finally, we solve Eq. (2.6), that is, Eq. (1.1) when $w=8$.

We observe that $\frac{1}{x} < \frac{3}{8} \Rightarrow x\geq2$.

Under the assumption $x\leq y\leq z$, Eq. (2.6) gives $\frac{1}{x} + \frac{1}{y} + \frac{1}{z} \leq \frac{3}{x}$ and thus $x\leq8$.

Hence $x\in\{2, 3,4,5,6,7,8 \}$ and thus Eq. (2.6) leads to the following equations:

For $x=2$,

2.41 $$ \frac{1}{x} + \frac{1}{y} =- \frac{1}{8}, $$

For $x=3$,

2.42 $$ \frac{1}{y} + \frac{1}{z} = \frac{1}{24}, $$

For $x=4$,

2.43 $$ \frac{1}{y} + \frac{1}{z} = \frac{1}{8}, $$

For $x=5$,

2.44 $$ \frac{1}{y} + \frac{1}{z} = \frac{7}{40}, $$

For $x=6$,

2.45 $$ \frac{1}{y} + \frac{1}{z} = \frac{5}{24}, $$

For $x=7$,

2.46 $$ \frac{1}{y} + \frac{1}{z} = \frac{13}{56}, $$

For $x=8$,

2.47 $$ \frac{1}{y} + \frac{1}{z} = \frac{1}{4}. $$

We avoid Eq. (2.41) because it leads to negative solutions.

Solving Eqs. (2.42)–(2.47) by the above procedure, we get:

$$\begin{aligned} ( x, y, z ) \in&{}\bigl\{ (3,25,600),(3,26,312),(3,27,213),(3,28,168),(3,30,120),(3,32,96), \\ &( 4,9,72 ), ( 4,10,40 ), ( 4,12,24 ), ( 4,16,16 ), ( 8,5,20 ), ( 8,6,12 ), ( 8,8,8 ), \\ & ( 5,6,120 ),( 5,8,20 ), ( 6,9,72 ),(6,10,40), (6,12,24),(6,16,16)\bigr\} . \end{aligned}$$

The above solutions ($w,x, y, z$) are found under the assumption $w\leq x\leq y\leq z$. Thus we can conclude that any permutation of ($w, x,y, z$) is a solution of Eq. (1.1).

We now state the following theorem which follows the above discussion.

### Theorem 2.1

*The equation* $\frac{1}{w} + \frac{1}{x} + \frac{1}{ y} + \frac{1}{z} = \frac{1}{2}$ *has only a finite number of solutions in the positive integers* $w, x, y$, *and* *z*.

We now state and prove general results.

### Theorem 2.2

*The Diophantine equation*

2.48 $$ \frac{1}{w} + \frac{1}{x} + \frac{1}{y} + \frac{1}{z} = \frac{m}{ n}, $$

*where* $m, n>1$ *are integers*, *has only a finite number of solutions in the positive integers* $w, x, y$, *and* *z*.

### Proof

Let us assume that $w \leq x \leq y \leq z$. Then

$$\begin{aligned} \frac{4}{z} &\leq \frac{1}{w} + \frac{1}{x} + \frac{1}{y} + \frac{1}{z} \leq \frac{4}{w} \\ &\Rightarrow \frac{4}{z} \leq \frac{m}{n} \leq \frac{4}{w} \\ &\Rightarrow \frac{1}{z} \leq \frac{m}{4n} \leq \frac{1}{w} \\ &\Rightarrow z\geq \frac{4n}{m} \geq w. \end{aligned}$$

Again $\frac{1}{w} < \frac{m}{n} $ and thus $w > \frac{n}{m}$.

This shows that $w\in ( \frac{n}{m}, \frac{4n}{m}]$ and hence *x*has only a finite number of integer values.

Now let $w = p_{1}$ be such an integer value. Then Eq. (2.48) gives

2.49 $$ \frac{1}{x} + \frac{1}{y} + \frac{1}{z} = \frac{m}{n} - \frac{1}{ p_{1}} = \frac{p_{1} m-n}{p_{1} n} = \frac{m_{2}}{n_{2}}. $$

Also, $x\leq y\leq z\Rightarrow \frac{3}{z} \leq \frac{m_{1}}{n_{1}} \leq \frac{3}{x} \Rightarrow x\leq \frac{3 n_{1}}{m_{1}} \leq z$.

But $x> \frac{n_{1}}{m_{1}}$ as $\frac{1}{x} < \frac{m_{1}}{n_{1}}$. Thus $x\in ( \frac{n_{1}}{m_{1}}, \frac{3 n_{1}}{m_{1}}]$ and hence *x*can take only a finite number of integer values. Let $x= p_{2}$ be such a value. Then Eq. (2.49) implies

2.50 $$ \frac{1}{y} + \frac{1}{z} = \frac{m_{1}}{n_{1}} - \frac{1}{p_{2}} = \frac{m_{2}}{n_{2}}. $$

Since $\frac{m_{2}}{n_{2}} \leq \frac{2}{y} $, so that $y\in [ p_{2}, \frac{2 n_{2}}{m_{2}} ]$ and thus *y*can also take only a finite number of integer values. Finally, if $y= p_{3}$ is such a value, then Eq. (2.50) gives $z= \frac{p_{3} n_{2}}{p_{3} m- n_{2}}$. Thus the number of integer values of *z* is also finite. □

Following a similar procedure, we can also establish the following result.

### Theorem 2.3

*The Diophantine equation*

2.51 $$ \frac{1}{x_{1}} + \frac{1}{x_{2}} + \frac{1}{x_{3}} +\cdots+ \frac{1}{ x_{n}} = \frac{p}{q}, $$

*where* $p, q >1$ *are integers*, *has only a finite number of solutions in the positive integers* $x_{1}, x_{2},\ldots, x_{n}$.

## Conclusion

In this paper, we explicitly find the solutions in positive integers $w, x, y$, and *z* of the title equation. Applying an analogue procedure, we prove that the Diophantine equation

$$\frac{1}{w} + \frac{1}{x} + \frac{1}{y} + \frac{1}{z} = \frac{m}{n}, $$

where $m, n>1$ are integers, has only a finite number of solutions in the positive integers $w, x, y$, and *z*. We finally claim that the same holds for Eq. (2.51).
